# A Pilot Randomized Controlled Trial Examining the Impact of Therapy Dog Visitation on Mood, Anxiety, and Depression in Patients Hospitalized for the Treatment of Mental Illness

**DOI:** 10.3390/healthcare14101420

**Published:** 2026-05-21

**Authors:** Nancy R. Gee, Lisa Townsend, Erika Friedmann, Sandra B. Barker, Tushar P. Thakre, Megan K. Mueller

**Affiliations:** 1 Center for Human-Animal Interaction, Department of Psychiatry, School of Medicine, Virginia Commonwealth University, Richmond, VA 23298, USA; lisa.townsend@vcuhealth.org (L.T.); sbbarker@vcu.edu (S.B.B.); tushar.thakre@vcuhealth.org (T.P.T.); 2 Department of Organizational Systems and Adult Health, University of Maryland School of Nursing, Baltimore, MD 21201, USA; friedmann@umaryland.edu; 3 Department of Comparative Pathobiology, Cummings School of Veterinary Medicine, Tufts University, North Grafton, MA 01536, USA; megan.mueller@tufts.edu

**Keywords:** animal-assisted intervention, mood, anxiety, depression, well-being, therapy dog, psychiatric inpatients

## Abstract

**Highlights:**

**What are the main findings?**

- Mood and anxiety improved more in the Animal-Assisted Intervention (AAI) condition than in either of the two control conditions.
- Males and people who live with a dog experienced greater improvements in the AAI condition.

**What are the implications of the main findings?**

- AAI is effective for reducing anxiety and depression and improving mood in those patients hospitalized for the acute treatment of mental illness.
- The conversational control (CC) condition did not consistently improve outcome measures, suggesting that there may be something special about the presence of a dog in this setting.

**Abstract:**

**Background/Objectives**: Evidence suggests that AAIs may be useful to support the mental health of individuals with psychiatric diagnoses, but there is limited research on the efficacy of AAIs for patients hospitalized for the treatment of acute mental illness. **Methods**: A randomized controlled trial (RCT) design, where patients hospitalized for the treatment of mental illness (N = 60) were randomized into to one of three conditions: dog + handler intervention (AAI), handler only conversational control (CC), or usual care (UC), for 20 min per day for three days. Mood (Smiley Face Assessment Scale), Anxiety (State Trait Anxiety Scales) and Depression (Center for Epidemiological Studies) were the outcome measures. Linear Mixed Models with random intercepts were applied to compare changes in anxiety and mood between conditions pre/post daily intervention session and pre/post three-day intervention phase (days 2–4), and anxiety and depression were assessed from baseline to the day after intervention (days 1–5) (p < 0.01). **Results**: Mood and anxiety improved more for the AAI than for the CC or UC conditions. Females and people who did not live with a dog experienced similar improvements in mood in the AAI and CC conditions relative to UC, while males and people who live with a dog experienced greater improvement in the AAI condition. Depression decreased similarly in the AAI and CC conditions and both were significantly better than the UC condition. **Conclusions**: These results indicate that a dog + handler interaction is effective for reducing anxiety and depression and improving mood in adults hospitalized for the treatment of mental illness. Human handler-only visits did not consistently result in similar findings, indicating that there may be something unique and beneficial about the presence of the dog.

## 1. Introduction

Psychiatric hospitalization represents a time of crisis for most patients. Individuals entering acute mental health care face symptom exacerbation and suicidality, both of which often interact with significant environmental stressors [1]. Engaging patients quickly in their treatment represents a key element of crisis stabilization. Research suggests that patient-centered, quality daily activities can facilitate connections between patients and staff and pave the way for a successful inpatient experience [2].

Symptom exacerbation (significant depressive symptoms, suicidality, anxiety and agitation) interferes with social connections, activities of daily living, and the ability to process information. Reducing symptoms at the outset of hospitalization eases transition to discharge and sets the stage for sustained post-hospitalization recovery. Research has explored adjunctive therapies to catalyze symptom reduction and strengthen patient engagement. These include mindfulness and meditative interventions [3,4] as well as truncated versions of standard therapies [5,6,7,8,9]. Many of these interventions require staff to deliver and monitor them, which can make these interventions difficult to incorporate into routine acute care.

Animal-assisted interventions (AAI) represent a low-cost adjunctive therapy that can be delivered by trained volunteers. Research on the impact of animals in mental health and medical settings is on the rise and tends to show that AAIs reduce negative outcomes, including anxiety [10]. The results of a meta-analysis conducted by Waite and colleagues [10] suggest that AAI can bring about large changes in outcomes such as anxiety, but it is important to note that the studies they reviewed included much variation in methodologies and measures used to assess outcomes. Though they reported large effect sizes, their findings suggest that AAI may only be superior to no treatment, and not as effective when compared to other social interventions, suggesting that their efficacy is due to the social interaction aspect of the intervention. Further, they call for a need for more randomized controlled trials in this research area.

A recent systematic review was conducted on 31 studies examining the impact of AAI on anxiety in vulnerable populations [11]. The research primarily involved dogs or horses and consistently showed the beneficial effects of AAI across populations such as pediatric hospitalization, chronic illness, disability, acute care, posttraumatic stress disorder and trauma exposure. The long-term outcomes were mixed, and the methodologies employed were variable, limiting cross-study comparisons.

### AAI in Psychiatric Settings

Evidence suggests that AAIs may be useful to support the mental health of individuals with psychiatric diagnoses during the rehabilitation process. Animal-Assisted Therapy (AAT) is a subtype of AAI that involves including an animal in goal-directed activities. Calvo et al. [12] conducted a small-scale randomized controlled trial (RCT) involving patients with schizophrenia in a rehabilitation program. The patients assigned to AAT as an adjunct to their rehabilitation program showed significantly greater improvement in negative symptoms, had greater adherence to treatment, and lower cortisol levels as compared to the control group. These results suggest that AAT is a worthwhile adjunct to the process of mental illness rehabilitation. Similarly, Mittly et al. [13] conducted an RCT in a rehabilitation center in Budapest. They also compared standard care with and without AAT and reported improved quality of life and anxiety in the AAT compared with the standard care condition. They propose that the application of AAT be considered more widely in health care settings as a useful adjunctive therapy in rehabilitation. An RCT comparing AAT to a control group reported that AAT improved happiness and quality of life in chronic psychiatric patients living in residential care homes [14]. These studies on AAT are relevant, but do not assess AAIs not involving goal-directed activities, nor do they assess psychiatric inpatients undergoing care for the acute treatment of mental illness.

To our knowledge, no studies have implemented an RCT to examine whether AAIs delivered by hospital volunteers improve patients’ symptoms during psychiatric hospitalization. Barker et al. [15] used a pre-post crossover design to compare anxiety levels measured by the State-Trait Anxiety Inventory (STAI) in 230 hospitalized psychiatry patients participating in single-group therapeutic recreation (TR) sessions involving AAI compared to a standard group TR session. AAI was delivered by a volunteer therapy dog owner. Significantly reduced anxiety levels after the AAI TR session were found for patients with psychotic disorders, mood disorders, and other disorders, and after the standard therapeutic recreation session for patients with mood disorders. There were no significant differences in the response to the two interventions. Like previous research, this study also does not separate the effect of the animal from their human handler because both are present at the same time during the experiment.

Depression is one of the most frequent and intransigent co-morbidities among patients who become psychiatrically hospitalized, making it an important treatment target. AAI shows some promise as an adjunctive treatment for depression, but more methodologically stringent research is needed. Hoffmann et al. [16] also used the STAI and a pre/post cross-over design with patients hospitalized with major depression. In a sample of 12 patients, they found that anxiety was significantly reduced following a visit with a dog when compared to when the patients did not receive a visit with a dog. The researchers used 30 min sessions in both cases (dog present/dog absent). The dog absent condition included the research assistant who chatted with the patients about their experiences with pets and their history. This study does not allow us to determine the impact of the presence of the human because the research assistant was present in both conditions. The study also did not examine depression as an outcome.

In another cross-over study of 35 psychiatric patients undergoing electroconvulsive therapy in an inpatient setting, Barker et al. [17] found a significant reduction in fear, but not anxiety or depression, when patients participated in a single 15 min AAI session delivered by a volunteer alongside their therapy dog, compared with a 15 min session with magazines prior to ECT. This study does not allow us to distinguish the impact of the human handler from that of the dog because both were present in the AAI session and both were absent in the control condition.

Although yielding some positive results, these studies have important limitations. The sample sizes in two of the studies are small and cross-over designs can create a bias in reporting because the manipulation of the dog present compared to the dog absent conditions is obvious to participants. Further, neither of these studies separates the effect of the human handler from that of the dog.

A meta-analysis of studies conducted with psychiatric inpatients and nursing home residents suggests medium effect sizes for the contribution of AAI to reductions in depression [18]. However, psychiatric inpatients represented only 40% of the sample and AAIs varied from 1 to 24 sessions delivered in different formats (individual vs. group) [18]. Nevertheless, findings support further exploration of the efficacy of AAIs for psychiatric inpatients who are experiencing symptoms of depression.

Mood states in individuals experiencing symptoms of depression are important treatment targets, given their association with social perceptions [19,20], quality of life [21], emotion regulation, and cognitive processes [22,23]. These functions influence patients’ engagement with treatment and their ability to learn and apply therapeutic tools. Limited evidence also supports the association of AAI with improved mood in patients with psychiatric diagnoses. Brown and colleagues [24] used a quasi-experimental pre/posttest design to examine exposure to AAI. They assessed both psychiatric inpatients and staff and found that negative mood was decreased and positive mood was increased following exposure to the therapy dog. This study suggests that exposure to a therapy dog may be beneficial for improving mood in both patients and staff on psychiatry inpatient units. The lack of appropriate control conditions indicates that a more rigorous RCT is required to determine whether the interaction with the dog or the social interaction with the dog’s handler is responsible for the improvements in mood.

The studies described above demonstrate that AAIs are associated with some improvement in mood and anxiety in psychiatric populations treated in inpatient, outpatient and rehabilitation/residential settings. All, however, have design limitations. There is a clear need for research utilizing more rigorous designs to examine the use of AAI, as delivered by volunteers and their therapy dogs, with individuals undergoing acute psychiatric hospitalization that specifically target mood, depression, and anxiety. These outcomes are critical in supporting overall treatment success for those patients.

The current study was designed to fill this gap and assess whether AAI with dogs, delivered by hospital volunteers, represents an effective adjunct to treatment that will promote aspects of recovery when initiated during acute psychiatric hospitalization. Further, this study addresses the question of whether it is the dog, the handler, or both that are the component(s) of AAI that improve mood, anxiety and depression in this setting. We also explored whether AAI sessions, and a series of several sessions, lead to improvement in anxiety, depression, and mood in psychiatric inpatients. Finally, we explore whether patient characteristics such as gender and dog ownership affect symptom response.

The primary outcome measure was mood, the secondary was anxiety and the tertiary was depression. Based on previous research, we hypothesized that the presence of the dog would improve all three outcomes relative to the usual care (control) condition. Further, based on the findings of Gee and colleagues [25,26,27], we hypothesized that the dog condition would result in steeper trajectories of change than the handler-only condition when they are compared with the usual care condition.

## 2. Materials and Methods

Note: the methods reported below have been published elsewhere [27], so there will be similarities in reporting, but the authors have made every attempt to avoid self-plagiarism. The previous manuscript examined the impact of AAI on loneliness during the RCT. All results and related figures presented in this manuscript may resemble those presented elsewhere, but are, in fact, based on different data and represent new findings. This study was registered with clinicaltrials.gov under the protocol number: NCT05089201, on 7 October 2021. https://clinicaltrials.gov/study/NCT05089201?term=NCT05089201&rank=1 (Accessed on 2 March 2026).

### 2.1. Ethics Approval

The Virginia Commonwealth University Institutional Review Board (HM20021567) reviewed and approved this study. Additionally, the Institutional Animal Care and Use Committee classified the study as “exempt” because the dogs involved in this study were not subjects of the investigation, and they were all privately owned/housed. The Local Human Rights Committee also reviewed this study due to the vulnerable nature of the participants and classified it as being implemented in accordance with applicable Human Rights Regulations.

### 2.2. Design

This study was an RCT in which individuals hospitalized for the treatment of mental illness were randomly assigned to one of three treatment conditions: (1) Animal-Assisted Intervention (AAI)—visits from a human handler and their registered therapy dog, (2) Conversational Control (CC)—visits from a dog handler alone, or (3) Usual Care (UC)—the usual high-quality care provided in this hospital setting. The independent variable was group assignment; each participant received one condition. Repeated outcome measurements were obtained from each participant before and after the intervention period and over the course of the 3-day intervention.

The study was conducted in three phases: Screening/Baseline (day 1), Intervention Delivery (days 2 to 4), and After-Intervention (day 5). The primary outcome variables were mood, anxiety and depression. The random assignment to groups was generated using randomizer.org (accessed on 19 September 2021). EF, who had no contact with participants, stratified the randomization by dog ownership status (owner vs. non-owner) and randomly assigned participants into the three conditions in blocks of six (instead of three, to improve concealment from experimenters). These assignments were placed into envelopes that were opened by NG and provided to the experimenter after the experimenter successfully enrolled a participant in the study, by experimenters. This procedure prevented experimenters from having awareness of participants’ group assignments prior to their enrollment in the study.

**Figure 1:**
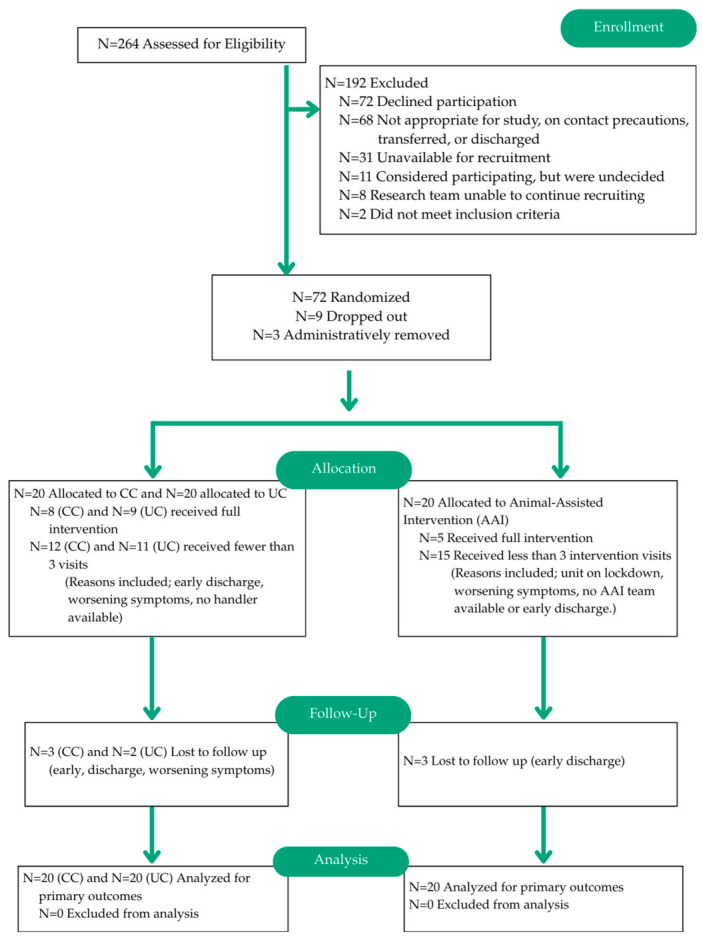
The study CONSORT flow diagram. The control conditions (CC and UC) are combined on the left side for simplicity of presentation.

The study CONSORT flow diagram is presented in Figure 1. This figure displays the AAI group information on the right side, with the two control conditions (CC and UC) combined on the left side of the diagram for simplicity of presentation. Blinding to the intervention group is not possible in a study of this nature because the participants and experimenters will be aware of the presence or absence of the dog. We refer the interested reader to previous discussions of methodological challenges involved in conducting this type of research [28,29]. No humans or dogs were harmed as a result of this study, and there were no adverse events to report (Supplementary Materials).

### 2.3. Therapy Dogs and Handlers

Sixteen privately owned/housed registered therapy dogs and their handlers were involved in the delivery of this study. Each of them met all requirements of the Center for Human–Animal Interaction Dogs on Call (DoC) program at Virginia Commonwealth University (VCU) (https://chai.vcu.edu/dogs-on-call/about-dogs-on-call/ Accessed on 2 March 2026). DoC is an evidence-based program [30] established in 2001, that has consistently provided therapy dog visitation to inpatients, outpatients, visitors, staff, and students throughout VCU and the VCU Health System (VCUHS). More details about the handlers, their dogs, and the DoC program can be found in Gee et al. [27] and details about program requirements can be found here: https://chai.vcu.edu/dogs-on-call/joining-dogs-on-call/ (Accessed on 3 March 2026).

### 2.4. Measures

#### 2.4.1. Screening

Individuals with cognitive impairment were excluded from the study based on concerns about difficulty in obtaining informed consent and the individual’s ability to respond to the study assessment instruments. The sole screening measure used in this study was the Brief Interview for Mental Status (BIMS) [31]. The BIMS is used in clinical settings to evaluate orientation, attention, and recall. It has good divergent and convergent validity and acceptable reliability (Cronbach’s alpha = 0.77). A score of 13 or higher is indicative of intact cognitive functioning [32]; participants who did not meet this threshold were excluded from the study.

#### 2.4.2. Demographics

Participants were asked to report basic demographic information, including gender, age, race/ethnicity, education, employment and marital status.

#### 2.4.3. Pet Ownership(s)

Participants were asked about current and previous pet ownership, including type and number of companion animal(s).

#### 2.4.4. Mood

Mood was measured using the Smiley Face Assessment Scale (SFAS) [33] which uses five emoticons to depict mood states ranging from very sad to very happy. This measure allowed us to quickly capture the patients’ mood state pre/post-intervention session. Given that this is used as a state mood measure, clinical cutoffs are not applicable. High scores indicate a more positive mood.

#### 2.4.5. Anxiety

Anxiety was measured using the 20-item State-Trait Anxiety Inventory (STAI-20) [34] and the 5-item version of the scale that was shortened for use with the State-Trait Anxiety Inventory for Adults (STAI-AD) [35]. The psychometric properties of both scales are well established and sound, with Cronbach’s alphas ranging from 0.82 to 0.94. Scores on the STAI-20 range from 20 to 80, with higher scores indicating greater anxiety, and a cutoff score of 40 or greater indicating probable clinical levels of anxiety. Scores on the shortened version are converted to the 20-item scale to reflect the same range and cutoff. Higher scores indicate greater anxiety.

#### 2.4.6. Depression

Depression was measured using the Center for Epidemiological Studies Depression 10-item short form (CESD), which has moderate to high reliability (0.45 to 0.81) and has been validated for use with hospitalized psychiatric patients [36]. The measure ranges from 0 to 30, with a clinical cutoff of 10 or above indicating depression. Higher scores indicate more symptoms of depression.

**Figure 2:**
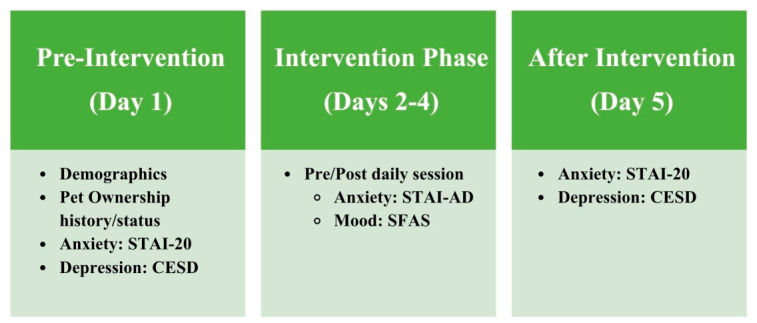
The timing and delivery of each measure used during the experiment.

The timing of delivery of each of the measures used in this study is displayed in Figure 2.

### 2.5. Procedure

The setting of this study was an adult psychiatry inpatient unit at a large university medical center in Virginia. This location includes two adult psychiatric units: a general unit and another designed for patients who may be aggressive and/or experience more extreme psychosis. This study took place in the general unit, which includes patients who do not pose a significant risk of aggressive behavior and whose symptoms do not prevent them from understanding instructions. Pre-intervention took place on day 1. On days 2–4, the intervention was delivered; participants completed daily pre-session measures prior to the 20 min intervention (AAI, CC, or UC) and completed daily post-session measures immediately after each intervention session. On day 5, all participants completed the after-intervention measures.

The AAI was designed to mimic typical therapy dog visits with patients in the hospital. Handlers were instructed to let the patients lead the conversation, but as is standard in the Dogs on Call program, they were given general topics of conversation in which to engage (e.g., dogs, the weather, sports, community events and activities) and topics they should avoid (e.g., politics, details of the patient’s treatment/condition). The experimenter waited outside the room, timing the intervention period. When 20 min were up, the experimenter knocked on the door and the handler politely ended the conversation (if any) and left the room with their dog. The same procedure was followed in the conversational control (CC, handler only) condition, except that the dog was not present. In the treatment as usual condition (UC) the experimenter waited outside the room, timing the intervention period. No attempt was made to alter or intervene in any ongoing treatment in any of the three intervention conditions.

In the AAI condition specifically, the patient was allowed to freely interact with the dog, touching and/or petting them if they initiated those activities. The patient was not instructed in how to interact with the dog, except that the handler was free to make comments such as “Wiley really likes to be scratched on his neck” or “Would you like him to come up on the bed with you?” The patient was free to decide when and how they interacted with the dog. The only element of control was the duration of the interaction, which was held to 20 min. In an effort to make these interactions as realistic as possible, we did not record or directly observe them, but handlers reported that in every case the patients did touch the dog.

Although each visit was individualized to meet the needs of the individual patient, there are a number of DoC program rules that all handlers were required to follow that restricted interaction options and made the visits relatively uniform overall. Some examples include: dogs were not allowed to shake hands with or lick people, they may not play fetch or perform trust falls, and they must be kept on a 4-foot (or shorter) leash at all times, restricting their range of movement. These restrictions and others related to the space and presence of sensitive equipment impact the type of interactions that are allowed or are possible with the parameters of the DoC program.

### 2.6. Recruitment

Study team personnel approached patients identified by clinical staff as meeting the inclusions criteria and provided them with information about the study. The team also made information packets available on the unit, so patients could request an opportunity to participate in the study. Eligibility requirements were assessed for a total of 264 patients to determine their eligibility to participate. At the time of enrollment, study personnel were blind to treatment group assignments. The enrollment rate for participants was 27.3%. The information regarding recruitment feasibility is reported elsewhere [37].

### 2.7. Participant Inclusion/Exclusion Criteria

Participant Inclusion Criteria: Aged 18+, English speaking, projected to be in the hospital 5 days (because the planned study required 5 days to implement; see Figure 2 above), ability to provide consent, and access to a phone after discharge. Participant’s ability to provide consent was assessed in three ways: (1) their BIMS score was 13+, (2) their status did not involve a legal guardian who would be required to provide consent on their behalf, and (3) in the clinical judgment of their healthcare team, they were fit to participate in the study. Participant exclusion criteria included (1) being COVID positive (COVID+) or on other contact precautions, and (2) having a fear or allergy related to dogs.

### 2.8. Informed Consent

This study included two stages of written or digital informed consent. In the screening consent stage, participants provided consent for an assessment of inclusion/exclusion criteria, which included taking the BIMS. Participants who met the inclusion/exclusion criteria then underwent the second stage of informed consent to participate in the study. All study participants completed both stages prior to participating in the study.

### 2.9. Participant Incentives

Participant incentives consisted of $20.00 USD gift cards. These cards were provided at three separate timepoints during the study: (1) Day 1, upon completion of all baseline measures; (2) 1 month later, upon completion of follow-up measures; and (3) 6 months later, upon completion of follow-up measures. If a participant completed all parts of the study, their total compensation was $60.00 USD. Note: return rates on all follow-up measures (at 1 and 6 months) were so low (valid data respectively for 1- and 6-month follow-up Ns were CESD; AAI = 0, 0, CC = 2, 2 and UC = 3, 3; STAI-20; AAI = 3, 2, CC = 0, 0, and UC = 2, 1) that the data were not usable and in the interest of brevity are not reported here.

### 2.10. Data Analysis

Initial descriptive analyses and assumption checking were performed. Based on the normality check, LMMS or generalized linear mixed models (GLMMs) with Poisson distributions and log links were used to examine differences in changes in outcomes among the intervention groups. For all analyses, participant identification number (ID) was included as a random intercept and intervention group was a three-level categorical independent variable that was converted into two dummy variables with UC as the reference group. These statistical approaches model intercorrelations among multiple measurements of the outcome and use maximum likelihood estimation. Thus, in longitudinal studies such as RCTs, these approaches do not require independence of successive measurements or equal numbers of assessments, allowing all data to be used [38]. Separate analyses were conducted for each outcome. Use of LMM/GLMM for data with dependence was confirmed by observation of high unconditional means intraclass correlation coefficients (ICCs) for all outcomes (ICCs: CESD = 0.81; STAI 20 = 0.69; SFAS = 0.57; STAI-AD = 0.73). Cohen’s d was calculated for effect sizes. Sequential Bonferroni corrections were used for the assessment of the significance of multiple comparisons as appropriate.

All analyses were conducted on an intent-to-treat basis. The use of LMM in an intention-to-treat analysis is considered the gold standard for the analysis of the results of clinical trials because it deals with missing data effectively [39].

The first set of analyses addressed changes during (from pre to post) the intervention sessions on days 2–4 of the study, the intervention phase. Predictors were pre-post, representing outcomes before and after the intervention sessions; day, representing the days within the intervention; group, representing the three randomly assigned intervention groups; the interactions of the intervention group with pre-post and with day; the interaction of day with pre-post; and the three-way interaction of day, pre-post, and intervention group. Two sets of stratified analyses were also conducted: one for individuals who lived with and without dogs at the time of hospitalization and one for women and men. The hypotheses were tested with the interaction of pre-post with the intervention group and day with the intervention group.

A second set of analyses was used to examine differences in mood, anxiety, and depression from day 1 to day 5. In these analyses, predictors were the intervention group dummy variables and time of assessment, as well as the interactions of the intervention group dummy variables with the time of assessment. The same two sets of stratified analyses were also conducted. The hypotheses were tested with the interaction of day with the dummy variables for the intervention groups.

### 2.11. Participants

Demographics for the sample of 60 participants are reported in Table 1. A sample size of 18 cases per group was based on a priori power calculations with power = 0.80, p = 0.05, Cohen’s d = 0.25, and within-subject correlation (0.7) [27]. Data collection and follow-up took place between 2 March 2022 and 17 April 2024. The sample consisted of 60 patients aged 18 to 73 years (M = 38.7, SD 17.8) who were residing in a psychiatric inpatient unit. A plurality of the sample was male (46.7%), approximately one third was black or African American, and 90% was non-Hispanic/Latino. More than half of the participants (51.7%) were single and more than half (64.9%) had at least some college education. Fewer than half of the participants were currently keeping a pet or currently keeping a dog. Additional demographic characteristics of the sample based on the group intervention condition are included in Table 2.

Analyses of Variance for continuous outcomes and Freeman–Halton extension of the Fisher exact test for 3 × 2 categorical variables [40] indicated that there were no significant differences in any of the baseline demographic characteristics among participants in the three intervention groups. There was no significant difference in odds of pet (43.3%) or dog (33.3%) ownership among the three intervention groups.

### 2.12. Baseline Outcomes

Table 2 displays participant characteristics according to intervention group. Participants’ baseline scores on the STAI-20 ranged from 20 to 80, which includes all possible scores on the scale. The mean anxiety score of 52.3 (SD = 14.40) is within the moderate anxiety range indicated by scores of 45 to 60. A score of 39–40 is generally considered the cut-point for clinically significant anxiety [34,35]. Baseline STAI-20 did not differ significantly according to intervention group.

Participants’ baseline CESD scores ranged from 13 to 38. All participants indicated some symptoms of depression as evidenced by the minimum score in the 10–15 range. The mean depression score for the participants was 26.5 (SD = 8.20) which is above the score of 21, which is considered the lower cutoff for the possibility of major depression. Baseline CESD scores did not differ significantly according to intervention group.

The first time mood was assessed, and the mean score was 2.55 (SD = 0.94), which is between somewhat sad (2) and neutral (3). There are no norms for the scale. Initial mood scores did not differ significantly among the intervention groups.

### 2.13. Intervention Phase Changes

After checking assumptions, linear mixed models (LMM) or generalized linear mixed models (GLMM) with random intercepts based on participant IDs were applied to examine differences in the changes in each outcome from the first to the last intervention session. Mood was examined with linear mixed models and anxiety (STAI-AD) was examined with GLMM. Predictors in the analysis included the interventions (AAI, CC, and UC) with AAI as the reference group; pre-post, representing the outcome scores before and after the intervention session; day, representing the day (1 to 3) of the intervention; and the interactions of the interventions with pre-post and within day. Exploratory stratified LMM and GLMM analyses were used to examine responses of subgroups, including males and females and dog owners and non-owners.

### 2.14. Study Duration: Baseline to Day 5

After checking assumptions, LMM with random intercepts based on participant IDs was applied to examine differences in the changes in anxiety and depression from baseline to the day after the intervention. The first set of analyses compared changes from baseline (Day 1) to after-intervention (Day 5). Predictors in the analyses included the intervention group (AAI, CC, and UC), the time variable (baseline to day 5), and the interaction of intervention group with the time variable. Stratified analyses also were conducted in a manner similar to those described above.

## 3. Results

### 3.1. Intervention Phase

As presented in the CONSORT diagram, while the complete intervention included three intervention sessions, participants completed one to three of the sessions. Table 3 displays the number of intervention sessions delivered to each participant by treatment condition. There was no significant difference in the distribution of the number of intervention sessions completed according to intervention group [Χ^2^(df = 4, N = 60) = 2.83, p = 0.59].

#### 3.1.1. Intervention Phase—Mood

**Figure 3:**
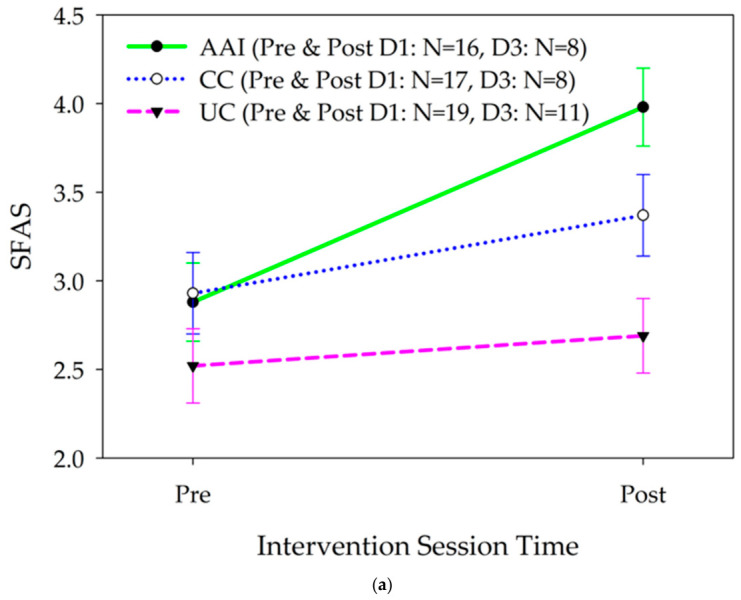

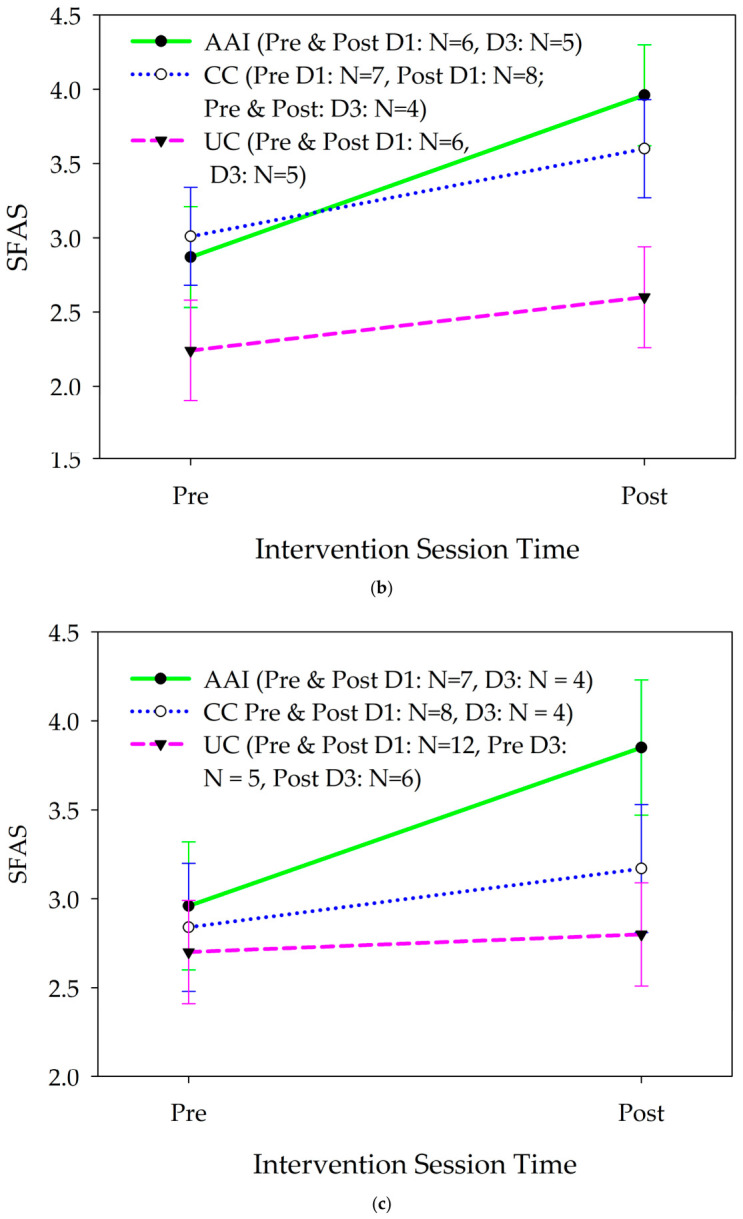

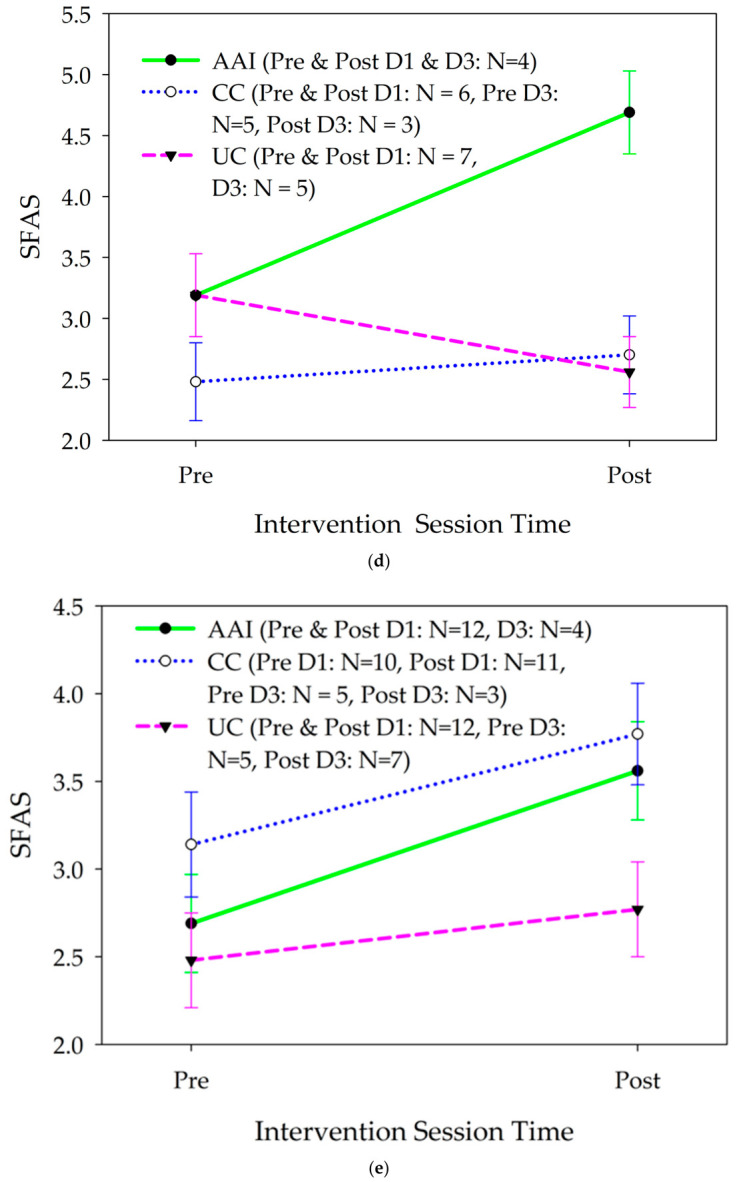
(**a**) Changes in mood (mean +/− sem) as assessed with the Smiley Face Assessment (SFAS) from before to after intervention sessions according to intervention group for all participants D1 = day 1, D3 = day 3 of intervention. (**b**) Changes in mood (mean +/− sem) as assessed with the Smiley Face Assessment (SFAS) from before to after intervention sessions according to intervention group for female participants. D1 = day 1, D3 = day 3 of intervention. (**c**) Changes in mood (mean +/− sem) as assessed with the Smiley Face Assessment (SFAS) from before to after intervention sessions according to intervention group for male participants. D1 = day 1, D3 = day 3 of intervention. (**d**) Changes in mood (mean +/− sem) as assessed with the Smiley Face Assessment (SFAS) from before to after intervention sessions according to intervention group for participants who lived with a dog. D1 = day 1, D3 = day 3 of intervention. (**e**) Changes in mood (mean +/− sem) as assessed with the Smiley Face Assessment (SFAS) from before to after intervention sessions according to intervention group for participants who did not live with a dog. D1 = day 1, D3 = day 3 of intervention.

Mood changed differently for the intervention groups from pre to post the intervention sessions [F(2283.04) = 10.82, p < 0.001; ES: AAI vs. UC = 0.77, AAI vs. CC = 0.53; Figure 3a]. Mood improved more for the AAI than UC group from before to after the intervention sessions. The trajectories of changes in mood from before to after the intervention sessions for the CC group were not significantly different from those for the AAI (b = 0.60, 95% CI = 0.23, 0.98). In analyses stratified by gender, there were differences in the trajectories of changes in mood according to intervention group from pre to post the intervention session for females [F(2, 110.6) = 3.69, p = 0.028, ES: AAI vs. UC = 0.66, AAI vs. CC = 0.44; Figure 3b] and for males [F(2, 124.7) = 3.83, p = 0.024, ES: AAI vs. UC = 069, AAI vs. CC = 0.48; Figure 3c].

For both men and women, the trajectory of improvement in mood from the beginning to the end of the intervention sessions was steepest in the AAI group [(Men: AAI vs. UC; b = 0.79, 95% CI = 0.22. 1.36; AAI vs. CC b = 0.56, 95% CI = −0.05, 1.16); (Women: AAI vs. UC: b = 0.73, 95% CI = 0.18, 1.27; AAI vs. CC: b = 0.50, 95% CI = −0.04, 1.05)]. In analyses stratified by living with a dog (yes/no), there were differences in the trajectories of changes in mood according to intervention group from pre to post the intervention session for participants who lived with a dog [F(2, 157.6) = 12.45, p = 0.049; ES: AAI vs. UC = 1.20, AAI vs. CC = 1.10; Figure 3d] and for those who did not [F(2, 98.0) = 16.93, p < 0.001; ES: AAI vs. UC = 0.51, AAI vs. CC = 0.21; Figure 3e]. In both participants who lived with and did not live with dogs, the trajectory of improvement in mood from the beginning to the end of the intervention session was steepest in the AAI group [(Lives with dog: AAI vs. UC: b = 1.42, 95% CI = 0.91. 1.93; AAI vs. CC b = 1.28, 95% CI = 0.73, 1.82); (Does not live with dog: AAI vs. UC: b = 0.59, 95% CI = 0.12, 1.06; AAI vs. CC: b = 0.25, 95% CI = −0.24, 0.74)].

**Figure 4:**
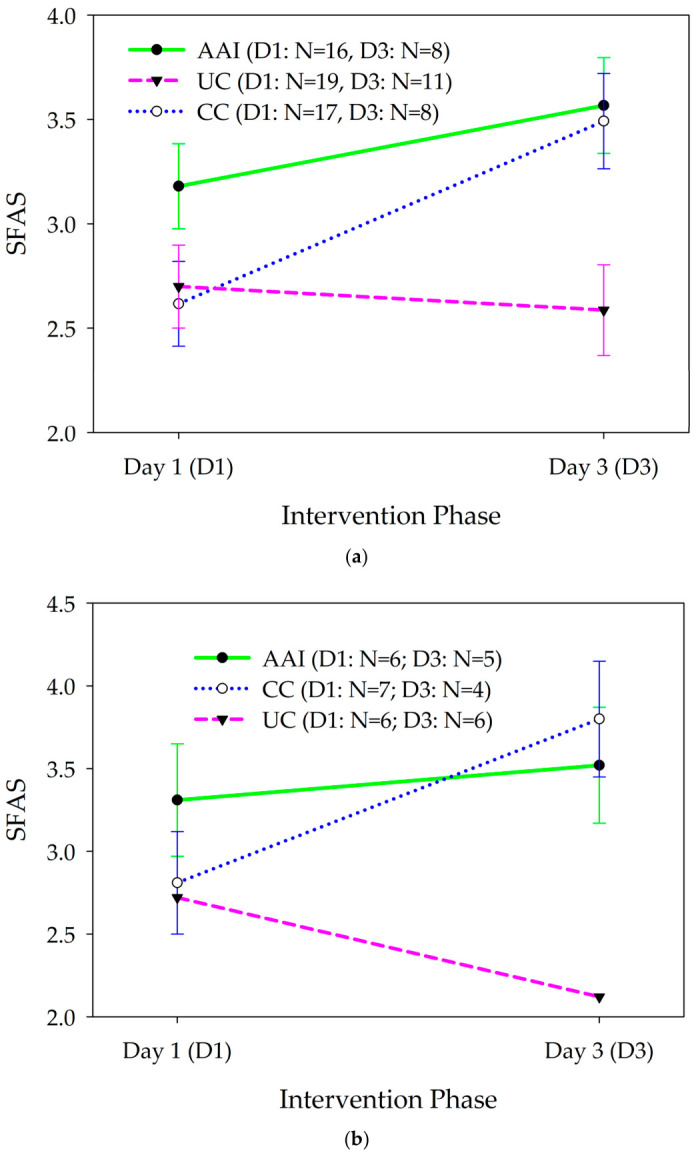

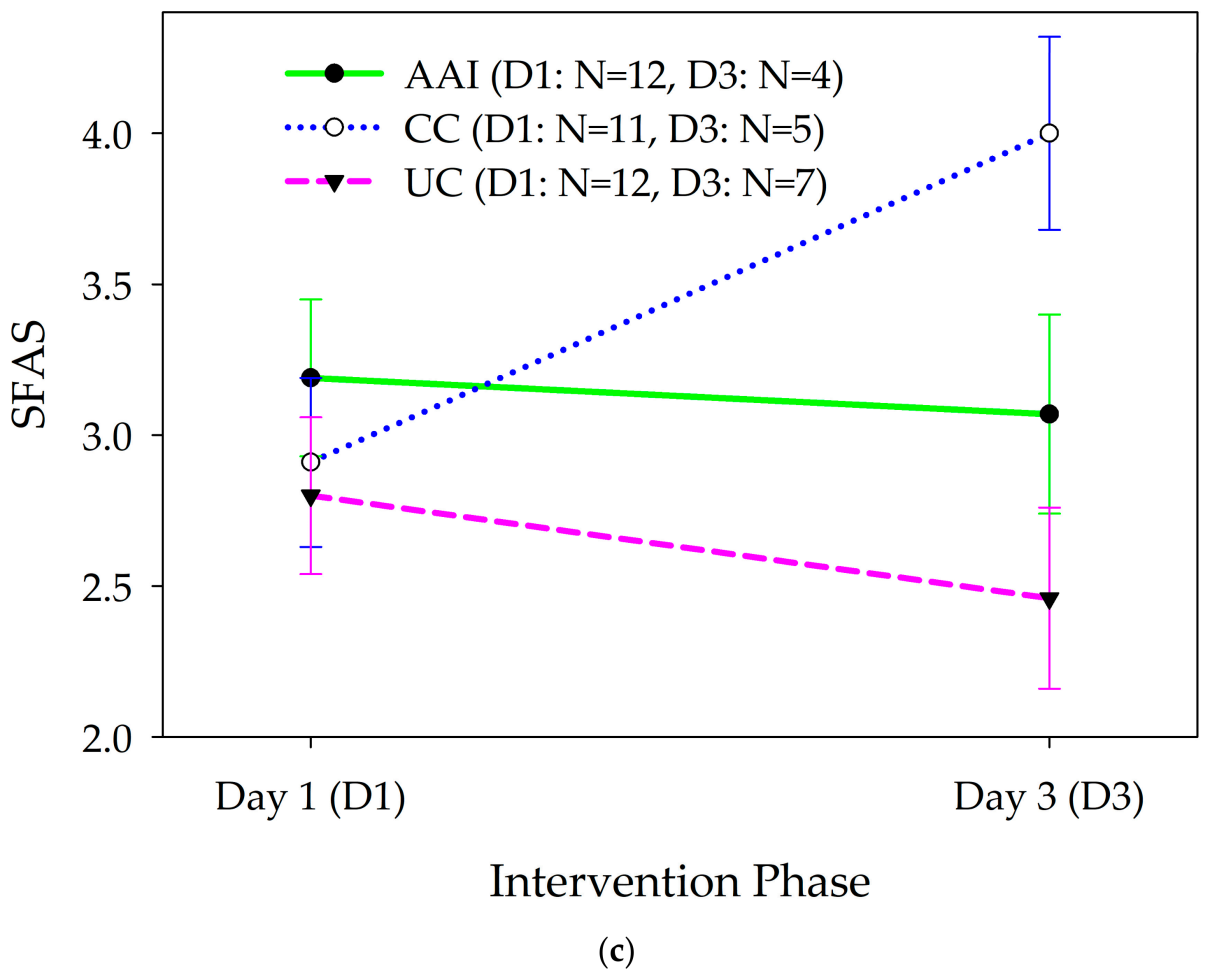
(**a**) Changes in mood (mean +/− sem) as assessed with the Smiley Face Assessment (SFAS) from day 1 to day 3 of the intervention phase according to intervention group among all participants. (**b**) Changes in mood (mean +/− sem) as assessed with the Smiley Face Assessment (SFAS) from day 1 to day 3 of the intervention phase according to intervention group among female participants. (**c**) Changes in mood (mean +/− sem) as assessed with the Smiley Face Assessment (SFAS) from day 1 to day 3 of the intervention phase according to intervention group among participants who did not live with dogs.

Mood also changed significantly differently for the intervention groups from the first to the last day of the intervention phase [F(2, 283.07) = 10.87, p < 0.001; ES: AAI vs. UC = 0.37, AAI vs. CC = −0.52); Figure 4a]. Mood improved significantly in both the AAI and CC intervention groups, but not the UC group. Mood improved more in the AAI than the UC group and more in the CC than in the AAI group (AAI vs. UC; b = 0.43, 95% CI = −0.03. 0.90; AAI vs. CC b = −0.60, 95% CI = −1.08, −0.11).

In stratified analysis, among women [F(2, 119.13) = 12.71, p < 0.001; ES: AAI vs. UC = 0.70, AAI vs. CC = −0.68; Figure 4b] and those who did not live with a dog [F(2, 179.2) = 12.45, 0 < 0.001, ES: AAI vs. UC = 0.19, AAI vs. CC = −1.06; Figure 4c], mood changed significantly differently from day 1 to day 3 of the intervention phase according to the intervention group. Among women, mood improved more from baseline to the day after the intervention phase in the CC intervention group than in the AAI or UC groups (AAI vs. UC; b = 0.81, 95% CI = 0.19, 1.43; AAI vs. CC b = −0.78, 95% CI = −1.40, −0.16). Among participants who did not live with dogs, mood improved more from baseline to the day after the intervention phase in the AAI group than in the CC or UC groups (AAI vs. UC; b = 0.22, 95% CI = −0.46, 0.90; AAI vs. CC b = −1.22, 95% CI = −1.89, −0.54). Mood did not change differently from baseline to the day after the intervention phase, according to the intervention group for men or for individuals who lived with a dog.

#### 3.1.2. Intervention Phase—Amount of Anxiety

**Figure 5:**
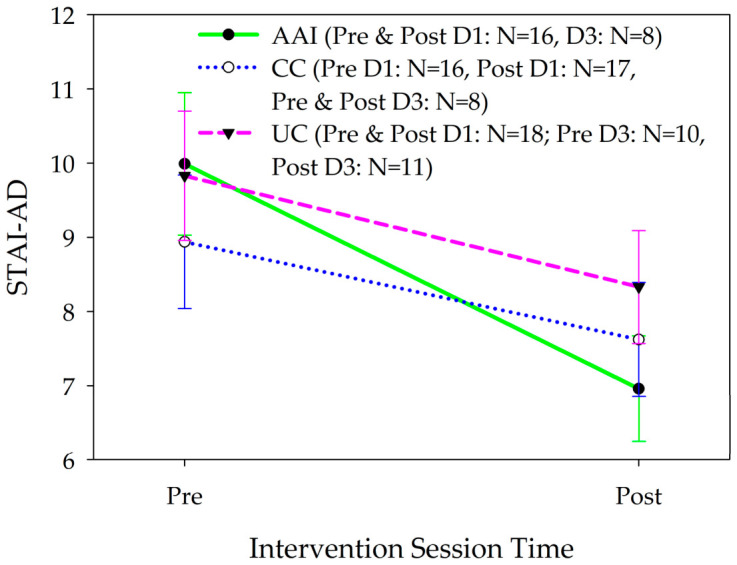
Changes in anxiety (mean +/− sem) as assessed with the 5-item State-Trait Anxiety Scale (STAI-AD) from before to after intervention sessions according to intervention group among all participants. D1 = day 1, D3 = day 3 of intervention.

In a GLMM, there were significant differences in changes in STAI-AD anxiety scores between the AAI and other groups from before to after the sessions [F(2252) = 3.354, p = 0.036; ES: AAI vs. UC = 0.28, AAI vs. CC = 0.24; Figure 5]. Anxiety decreased significantly more from the beginning to the end of the intervention sessions among participants in the AAI than the other intervention groups [AAI vs. UC: b(exp) = 1.00, 95% CI = 0.83, 1.19; AAI vs. CC: b(exp) = 0.86, 95% CI = 0.72, 1.03]. Neither analysis stratified by gender or by living with a dog revealed any differences in changes in anxiety from day 1 to day 3 of the intervention phase among the intervention groups.

### 3.2. Study Duration

#### 3.2.1. Baseline to Day 5—Amount of Anxiety

In an LMM there were no significant differences in the changes in STAI-AD anxiety scores among the intervention groups. Anxiety decreased significantly from baseline to day 5 [F(1, 53.77) = 8.00, p = 0.007; ES = 0.58; b = 8.52; 95% CI = 0.78, 16.25] and did not differ among the groups. In analyses stratified by gender and stratified by living with a dog, there were no significant differences in changes in anxiety from baseline to the day after the intervention according to intervention group in any of the subgroups.

#### 3.2.2. Study Duration: Baseline to Day 5 (Post-Intervention)—Depression

**Figure 6:**
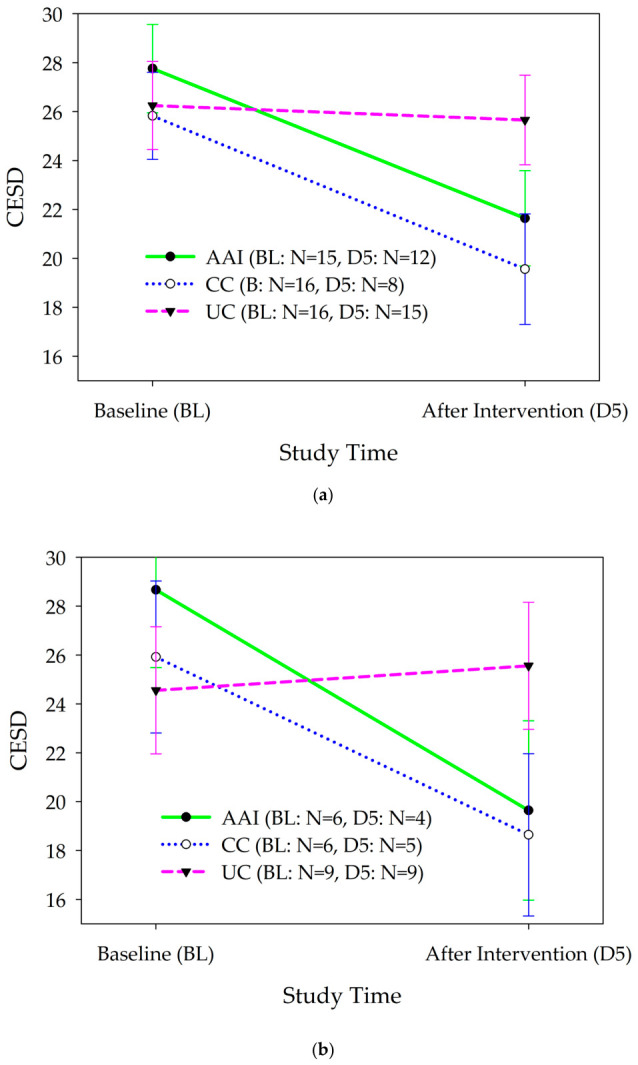
(**a**) Changes in depression as assessed with the Center for Epidemiological Studies Depression Scale (CESD) from baseline, the day before the intervention phase, to the day after the intervention phase according to intervention group among all participants. (**b**) Changes in depression (mean +/− sem) as assessed with the Center for Epidemiological Studies Depression Scale (CESD) from baseline, the day before the intervention phase, to the day after the intervention phase, according to intervention group among male participants.

There was a significant difference in the change in depression from baseline to the day after the intervention phase (day 5) according to the intervention group [F(2, 37.5) = 3.66, p = 0.035; ES: AAI vs. UC = 0.72, AAI vs. CC = 0.03; Figure 6a]. Depression decreased more in the AAI than in the UC group (b = −10.02, 95% CI = −18.69, −1.36). There was no significant difference in decreases in depression between the AAI and CC groups (b = 0.20, 95% CI = −5.42, 5.82). In analyses stratified by gender, among men, depression changed differently from baseline to the day after the intervention according to intervention group [F(2, 18.32) = 18.32, p = 0.04; ES: AAI vs. UC = −1.30, AAI vs. CC = 0.22; Figure 6b]. Depression decreased more in the AAI than in the UC group (b = −5.2, 95% CI = −10.35, −0.69). There was no significant difference in decreases in depression between the AAI and CC groups (b =−1.69, 95% CI = −11.55, 8.18). Depression decreased significantly from baseline to the day after the intervention phase among male participants in the AAI and CC intervention groups but not the UC group. Among women, participants who lived with a dog, and participants who did not live with a dog, there were no significant differences in changes in depression symptoms from baseline to the day after the intervention according to the intervention group.

## 4. Discussion

The results of this pilot RCT indicate that a dog-plus-handler interaction may be effective for reducing anxiety and depression and improving mood in adults hospitalized for the treatment of mental illness. Human handler-only visits did not consistently result in similar findings, indicating that there may be something unique and beneficial about the presence of the dog. Each outcome is presented and discussed separately below.

### 4.1. Mood

The results showed that mood improved more for the AAI condition than it did for the CC condition when both were compared to the UC condition pre- to post-daily intervention. When this analysis was stratified separately by gender and by dog cohabitation status, both men and women, as well as people who live with a dog and those who do not, experienced the steepest trajectories of improvement in mood in the AAI condition, suggesting that gender and dog ownership do not differentially impact the trajectory of improvement in mood from pre- to post-intervention. However, when the analysis examined the data from day 1 of the intervention to day 3, mood improved in both the AAI and CC intervention groups, but not in the UC group. In this case, mood improved more in the CC than in the AAI condition. This finding is inconsistent with other findings indicating faster trajectories of improvement in the AAI conditions. It is possible that the AAI effects are immediate but not long-lasting, whereas the effects associated with the CC condition may be cumulative over the three-day intervention. Likewise, over the course of the intervention period, when the analysis was stratified, women experienced greater improvements in mood in the CC condition than in the AAI or UC conditions, but people who do not live with a dog experienced greater improvements in mood in the AAI condition than in the CC or UC conditions. These results are unexpected because previous research has demonstrated greater improvement in mood in the AAI condition across genders [26]. It is possible that the female patients connected more with the primarily female dog handlers in the CC condition, creating a greater improvement in mood relative to their male counterparts. It was also unexpected that non-dog-owners would experience greater improvement in mood than dog-owners, but it is possible that when a dog owner gets a visit from another dog that reminds them of how much they miss seeing their own dog, which could offset the improvement in mood experienced by the non-dog-owners. A recent systematic review of the literature [41] reported a relationship between pet attachment and mood, so it is not surprising that being reminded of one’s own pet may have an impact on the participant’s overall mood, which could attenuate or overshadow the impact of a visiting therapy dog on their mood.

### 4.2. Anxiety

Anxiety decreased significantly more in the AAI condition from pre to post daily intervention session than in the CC or UC conditions. Stratified analyses did not reveal any significant differences in these findings based on gender or dog-ownership status, suggesting that AAI was globally effective at decreasing anxiety. This finding is noteworthy and indicates that AAI may be effective in producing short-term reductions in anxiety for people who are hospitalized for the acute treatment of mental illness. This suggests that AAI can serve as an effective and immediate adjunct to the treatment of anxiety in this population.

When changes in the amount of anxiety from day 1 to day 5 were examined, all groups experienced significant decreases in anxiety, and there were no significant differences in these changes based on group assignment, gender or dog-ownership. A general finding of reduced anxiety in this population is not surprising, given the fact that all patients were receiving mental health treatment with the goal of improving this outcome measure. The absence of an AAI benefit in this comparison suggests that we consider the possibility that the AAI influence is short-lived and immediate, rather than enduring, as has been found in other studies (e.g., [25,26,27]). Future research is needed to establish the timing and durability of AAI effects on anxiety in this population.

### 4.3. Depression

Depression decreased significantly from baseline to day 5 in the AAI and CC conditions, but it did not change in the UC condition. This finding is interesting because it suggests that the dog and perhaps the associated social interaction of the handler provided an effective adjunct to the ongoing mental health treatment of depression. In this case the presence of the dog with the handler did not improve the outcome relative to the handler only condition, suggesting that the presence of the handler may have been enough. Further, this result may be driven by the male participants who experienced this improvement in depression, whereas the female participants, whether they lived with a dog or not, did not experience similar changes in depression from baseline to day 5. Previous research has indicated a lengthier time course and higher chronicity of depression in females than in males [42]. We suspect that our short-term intervention may not have been potent enough to effect change for female participants during the study timeframe, but further research is needed to address this potential explanation. Additionally, gender differences in response to depression treatment have been described in the pharmacological literature [43,44], providing support for our speculation that our findings similarly reflect gender differences in response to depression treatment.

Although improvements were found across all three outcome measures, short-term improvements in anxiety and mood provided the clearest evidence supporting the use of AAI for patients hospitalized for the treatment for acute mental illness. This may be clinically significant given intense emotions (e.g., anxiety, sadness) may interfere with patients’ willingness to engage in acute care interventions, such as group or individual therapy. Furthermore, emotion dysregulation can substantially impair information processing, such as retention of coping skills training or the content of psychotherapy sessions [45]. In this sense, even transient improvements in anxiety, depression, and mood might enhance inpatients’ ability to engage with and benefit from standard acute care treatments.

Depression generally improved across the patient’s hospital stay regardless of intervention condition, which is not unexpected given the purpose of their hospitalization, but further investigation to determine if or when AAI may impact depression in patients hospitalized for the treatment of mental illness. We can speculate that AAI may have a smaller impact on depression in general, or that depression in those people hospitalized for the acute mental illness requires a larger effect size to see significant change.

Based on the results of a meta-analysis, Waite and colleagues [10] suggest that AAI may not be effective in improving mental health outcomes when compared to an appropriate social control intervention. The results of previous research [25,26,27] have consistently shown that the AAI condition creates positive trajectories of change in outcome measures (including mood and anxiety), whereas the CC condition is seldom significantly better than the UC condition, indicating that there is something special about the presence of the dog above and beyond the social contribution of the dog’s handler. This basic finding was born out in the anxiety outcome measure in the current study, but not for the other two measures. Depression generally improved across the days of the study, without significant changes based on condition, but mood provided more nuanced findings suggesting that gender and dog ownership status are important variables for future researchers to examine.

The findings of this study are consistent with the application of the biopsychosocial model for understanding the role of adjunctive interventions including HAI in maintaining mental health [46]. HAI specifically has been conceptualized as a social support that can impact psychological health directly or act indirectly through changes in physical aspects of health [47]. Specific mechanisms for AAI causing improvements in anxiety and mood need to be fleshed out. It is possible that because emotion processing and regulation are often impaired in psychiatric inpatients [48], we can speculate that exposure to a dog while in the hospital may improve emotion regulation via mechanisms such as stress reduction, distraction, or social lubrication. Other possible explanations exist. For example, the presence of the dog may reduce stress, or it may increase available oxytocin, or it may fulfill a fundamental drive to be in or with nature (e.g., biophilia) but further research is needed to tease out the specific mechanisms of action, operationalize variables that accurately represent theoretical constructs, and drive theory development and refinement.

### 4.4. Limitations

This study used a convenience sample of patients from an inpatient psychiatry unit. These individuals may or may not represent the larger population of all patients hospitalized for the treatment of mental illness. We did not restrict our sample to specific diagnoses, nor did we capture or analyze the data separately based on diagnosis. Our study was not intended to examine the impact of AAI on specific diagnoses. All participants were psychiatric inpatients undergoing individualized treatments, including medications and therapy. We made no attempt to record or manipulate those aspects of their ongoing treatment, so it is unknown if medications or therapy might have differentially altered outcomes for one or more of the treatment groups. The randomization process should have distributed any related effects equally across the three treatment groups.

To our knowledge, there is no way to conduct AAI research in a double-blind manner. All participants who received the dog intervention were aware of the presence of the dog and they interacted with that dog. Likewise, experimenters were also aware of the presence of the dog. Additionally, the use of industry-standard self-report measures to assess the outcomes of interest in this study may be subject to expectancy effects due to the lack of blinding. However, given that participants only received one arm of the study (AAI, CC or UC), it is unclear how they might have a higher expectancy in any individual condition relative to the other conditions. However, if their expectancy was higher in the AAI condition, one could argue that expectancy may be integral to the benefits found from interacting with a dog. It is possible that expecting one’s anxiety to be reduced in the presence of a dog is a mechanism driving the effect. We leave it to future researchers to find a way to disentangle expectancy effects from the effects of interacting with a dog.

This pilot study used a sample that was too small to make certain interesting statistical comparisons (e.g., examining specific diagnoses or those individuals who classified their gender as non-binary) that would require dividing the existing data into even smaller segments. It is also possible that a Type II error could mask the presence of other potentially significant findings in the subgroup analyses that were conducted. Linear mixed models use maximum likelihood estimates to allow analysis of all available data, and there was some attrition during the intervention and from day 1 to 5, which may have resulted in some null findings in the study. A larger sample size is required to address this potential limitation. Also, because the return rates were too low on the one- and six-month follow-up measures, we are unable to assess or report on the effects of the AAI over time. Future research is needed to examine the time course of the effects reported here.

Although we used the same intervention protocol as was used in other studies (e.g., [25,26,27]), it is possible that it does not generalize to other types of dog–human interactions, AAI programs, different visitation types, or visitation durations. We involved 16 different dog–handler pairs in the study, but the sample was too small to determine if there were dog/handler effects. It is possible that people will respond differently to different dog/handler teams, and as such, we recommend that future researchers investigate this possibility. It is also possible that handler characteristics (i.e., gender) or dog characteristics (i.e., size, color) as well as participant characteristics are related to the efficacy of the AAI for specific outcomes. This possibility is worth exploring in future larger scale RCTs.

## 5. Conclusions

This pilot study offers a number of strengths, including the implementation of an RCT, use of measures with sound psychometric properties, standardized intervention protocol that replicates and extends previous work ([25,26,27]), high standards of ethical treatment of humans and dogs, and best practice delivery of a dog-visitation process in a hospital setting. The results of the current study replicate and extend previous work and support the conclusion that AAI may be effective in the short-term improvement of anxiety and mood in patients hospitalized for the acute treatment of mental illness. This study also demonstrates that the presence of the dog is unique and impactful above and beyond the presence of their handler. These findings suggest that AAI, involving dogs and delivered by volunteers, is a beneficial adjunct to treatment in this setting.

This study points to the need for well-powered multi-site RCTs that include information about patient diagnoses, medications, and ongoing therapeutic treatments, in addition to the use of both self-report and physiological measures. Further, we depend upon such studies to flesh out the optimal dosage of AAI, the time-course of effects related to AAI, as well as to provide a clear theoretical understanding of the mechanisms driving these effects.

## Figures and Tables

**Table 1:** Participant demographic characteristics; N = Number, % = Percent, M = Mean, and SD = Standard Deviation.

	N	%	M	SD
Age (years)			38.7	17.8
Baseline Anxiety			52.3	14.4
Baseline Depression			26.7	8.2
Baseline Quality of life			2.5	5.0
**Gender**				
Female	26	43.3		
Male	28	46.7		
Non-binary	6	10.0		
**Race**				
White	34	56.7		
Black/African American	20	33.3		
Asian	3	5.0		
American Indian/Alaskan Native	2	3.3		
Ethnicity				
Not Hispanic/Latino	54	90.0		
Hispanic/Latino	2	3.3		
**Marital Status**				
Single	31	51.7		
Married/long-term partnership	17	28.3		
Divorced	8	13.3		
Widowed	3	5.0		
**Education**				
Less than high school	4	6.7		
High school/GED	17	28.3		
Some college	20	33.3		
Bachelor’s degree	11	18.3		
Graduate degree	8	13.3		
**Current Pet Ownership**				
Yes	26	43.3		
No	32	53.3		
**Current Dog Ownership**				
Yes	20	33.3		
No	40	66.7		

**Table 2:** Participant demographic characteristics according to intervention group: Animal-Assisted Intervention (AAI), Conversational Control (CC), Usual Care (UC), N = Number, % = Percent, M = Mean, and SD = Standard Deviation.

Group	AAI	CC	UC	
	N (%)	N (%)	N (%)	Exact p
Female	8 (40.0)	11 (55.0)	7 (35.0)	0.44
Married/long-term partnership	5 (26.3)	5 (25.0)	7 (35.0)	0.81
High school/GED or less	7 (35.0)	7 (35.0)	7 (35.0)	1.00
White	13 (68.4)	10 (50.0)	11 (55.0)	0.48
Not Hispanic/Latino	17 (94.7)	19 (100.0)	18 (94.4)	0.77
Current Pet Owner	7 (36.8)	10 (52.6)	9 (45.0)	0.64
Current Dog Owner	5 (25.0)	7 (35.0)	8 (40.0)	0.70
	M (SD)	M (SD)	M (SD)	F(df), p
Age	36.5 (17.4)	39.9 (18.5)	39.6 (18.3)	0.22 (2, 55), 0.80
CESD Baseline	27.2 (7.2)	26.6 (6.7)	26.2 (4.7)	0.90 (2, 44), 0.92
STAI-20 Baseline	52.8 (16.8)	51.3 (15.2)	52.7 (11.8)	0.07 (2, 53), 0.94
SFAS First Assessment	2.7 (1.1)	2.4 (0.8)	2.6 (0.9)	0.91 (2, 99), 0.40

**Table 3:** Number of intervention sessions delivered by treatment condition (AAI = Animal-Assisted Intervention, CC = Conversational Control, UC = Usual Care).

	1 Session	2 Sessions	3 Sessions	Total
AAI	9	6	5	20
CC	8	4	8	20
UC	6	4	10	20
Total	23	14	23	60

## Data Availability

The data presented in this study are available via Open Science Framework at: https://osf.io/q4dzc (Accessed on 2 March 2026).

## Supplementary Materials

The CONSORT 2025 [49] checklist can be downloaded at: https://www.mdpi.com/article/10.3390/healthcare14101420/s1.

## Abbreviations

The following abbreviations are used in this manuscript:

AAI	Animal-Assisted Intervention
AAT	Animal-Assisted Therapy
BIMS	Brief Interview for Mental Status
BL	Baseline
CESD	Center for Epidemiological Studies Depression 10-item short form
COVID	Coronavirus Disease
COVID+	COVID positive
CC	Conversational Control
CI	Confidence Interval
DoC	Dogs on Call
D1	day 1
D2	day 2
D3	day 3
ES	Effect Size
GLMM	Generalized Linear Mixed Models
ICC	Intraclass Correlation Coefficients
ID	Participant identification number
LMM	Linear Mixed Models
RCT	Randomized Controlled Trial
Sem	standard error of the mean
SFAS	Smiley Face Assessment Scale
STAI-20	State-Trait Anxiety Inventory
STAI-AD	State-Trait Anxiety Inventory for Adults
UC	Usual Care
VCU	Virginia Commonwealth University
VCUHS	VCU Health System
